# Agroprospecting of Biowastes: Globe Artichoke (*Cynara scolymus* L. Cultivar *Tema*, Asteraceae) as Potential Source of Bioactive Compounds

**DOI:** 10.3390/molecules29163960

**Published:** 2024-08-22

**Authors:** Jorge M. Alves-Silva, Mónica Zuzarte, Lígia Salgueiro, Emma Cocco, Valentina Ghiani, Danilo Falconieri, Delia Maccioni, Andrea Maxia

**Affiliations:** 1 Faculty of Pharmacy, University of Coimbra, Azinhaga de S. Comba, 3000-548 Coimbra, Portugal; jmasilva@fmed.uc.pt (J.M.A.-S.); mzuzarte@uc.pt (M.Z.); ligia@ff.uc.pt (L.S.); 2 Faculty of Medicine, Coimbra Institute for Clinical and Biomedical Research (iCBR), University of Coimbra, Azinhaga de S. Comba, 3000-548 Coimbra, Portugal; 3 Department of Chemical Engineering, Chemical Engineering and Renewable Resources for Sustainability (CERES), University of Coimbra, 3030-790 Coimbra, Portugal; 4 Laboratory of Economic and Pharmaceutical Botany, Department of Life and Environmental Sciences, University of Cagliari, V.le S. Ignazio da Laconi 13, 09123 Cagliari, Italya.maxia@unica.it (A.M.); 5 Autonomous Region of Sardinia, 09124 Cagliari, Italy

**Keywords:** agroprospecting, *Cynara scolymus* L., Tema *cultivar*, biowaste, skin aging, senescence

## Abstract

Artichokes (*Cynara scolymus* L.) are valuable foods, thanks to their health benefits, but they generate significant waste during their production, harvesting, and processing, which poses sustainability issues. This study applied an agroprospecting approach to convert Tema artichoke biowaste (TB) into valuable resources, starting from a global perspective of the production chain to the targeted applications based on chemical and biological analysis. The major TB was identified in the outer bracts of the immature flower heads, which were collected throughout the harvesting season, extracted, and analyzed. The most abundant compounds were phenolic acids including chlorogenic acid and caffeoylquinic derivatives. Among flavonoids, cynaroside was the most abundant compound. Multivariate analysis distinguished batches by collection period, explaining 77.7% of the variance, with most compounds increasing in concentration later in the harvest season. Subsequently, TB extracts were analyzed for their potential in wound healing and anti-aging properties. Fibroblasts were used to assess the effect of selected extracts on cell migration through a scratch wound assay and on cellular senescence induced by etoposide. The results show a significant decrease in senescence-associated β-galactosidase activity, γH2AX nuclear accumulation, and both p53 and p21 protein levels. Overall, this study ascribes relevant anti-skin aging effects to TB, thus increasing its industrial value in cosmeceutical and nutraceutical applications.

## 1. Introduction

Agricultural waste, such as crop residues, peels, leaves, and roots, constitutes a significant portion of the organic waste produced, with an estimated 2800 to 3800 million tonnes/year globally [1]. Without proper disposal or reutilization, this waste can significantly contribute to soil and water pollution and can emit greenhouse gases during decomposition [2]. Therefore, it is essential to develop effective strategies for reusing and valorizing this waste, transforming it into valuable resources like compost, biogas, or ingredients for new food products or animal feedstock [3].

In addition to greater environmental sustainability, the transition to a circular food system brings direct economic benefits through the valorization of waste and indirect benefits through the expansion of the production chain [4].

With this work, the authors aim to address the agricultural waste problem by implementing a new way of thinking about crops, summarizing this type of approach as “Agroprospecting”. In this context, the term agroprospecting refers to the exploration of agrobiodiversity co-products and organic waste, which are from intensive, extensive, local, regional, traditional, or technological crops, in search of small- or macro-molecules that could be developed into materials of commercial value for agriculture, bioremediation, cosmetics, nanotechnology, or pharmaceutical and food industries. Agroprospecting (agro-exploration) is fundamental to closing the loop into a circular and sustainable food system; it analyzes the production chain and assesses the potential reuse, recycling, or transformation of organic residues, thus minimizing waste production.

Leveraging the principles of agroprospecting, the present study focuses on the artichoke cultivar Tema (*Cynara scolymus* L., Asteraceae) and addresses the considerable waste typical of artichoke cultivation; we aim to transform these byproducts into valuable co-products within a sustainable circular economy.

Tema cultivar is a commercial artichoke hybrid that represents a variety of Violetto artichoke and is characterized by a cylindrically shaped head lined with purple bracts ending in a short thorn [5]. The bracts are fleshy and tender with little bitter flavor. Its excellent cold resistance allows an extended harvesting period from November until March, depending on the year. Notably, this cultivar showed good resistance to the browning processes [6], a feature that makes it suitable for obtaining a fourth-range product.

However, due to the processing steps, it is characterized by a large amount of waste, namely the leaf portions of the stem and especially the outermost bracts. These aspects are common in artichoke cultivation; in fact, the edible portion of the artichoke is the immature flower head, which undergoes further processing to remove the outer bracts, leaves, and stems. Additionally, waste production in the artichoke’s production chain increases due to common agricultural practices involving the removal of secondary and tertiary flower heads. At the end of processing, approximately 80–85% of the total biomass is discarded [7]. Given the high waste generation, intensive processing, low yields, and significant field requirements (such as pesticides, fertilizers, and irrigation), artichoke cultivation is considered one of the least environmentally sustainable agricultural practices. This results in significant greenhouse gas emissions, and recent studies have calculated that the carbon footprint along the production chain is around 8000 kg CO_2_eq per hectare [8].

Despite growing concerns about the environmental footprint of the artichoke supply chain due to high waste generation and resource requirements, the Mediterranean basin hosts the largest global production of artichokes, primarily in Egypt, Italy, and Spain [9]. Italy ranks as the world’s second-largest producer, with 378,110 tonnes in 2022, corresponding to a cultivated area of approximately 38,170 hectares [10]. Furthermore, Italy is recognized as the “primary cultivated gene pool” due to its significant reservoir of autochthonous germplasm. Numerous recognized cultivars exhibit variations in characteristics such as morphology (e.g., Spinoso, Romanesco, Catanese, and Violetto) or production period, which enables early production in autumn–winter and late production and allows harvesting until spring. Furthermore, ongoing research, which focuses on new hybrid varieties propagated from seed, has been introduced; these include Tema, Apollo, Madrigal, and Concerto [11,12].

In addition, artichoke cultivation offers valuable health benefits through its content of fibers and bioactive compounds like phenolic acids, terpenes, and flavonoids, which contribute to its status as a potent functional food [13,14,15,16]. Collectively, these compounds reduce the inflammatory processes, regulate sugar and fat metabolism and digestive processes, and decrease the risk of chronic diseases [17,18,19]. Among its primary bioactive molecules are chlorogenic acid, cynarin, and cynaropicrin, along with various isomers of caffeoylquinic acid and glycosidic derivatives of luteolin and apigenin [17,20,21].

In particular, chlorogenic acid is associated with antidiabetic and antilipidemic effects; it helps regulate blood glucose levels by modulating glucose absorption and metabolism, contributing to lipid-lowering and cholesterol-lowering actions that improve cardiovascular health by managing lipid profiles and reducing the risk of atherosclerosis [22,23]. Another fundamental bioactive metabolite is the sesquiterpene lactone cynaropicrin. Despite its low concentration, it is reported to possess robust anti-inflammatory effects by inhibiting pro-inflammatory cytokines and modulating immune responses, partly through suppression of the key pro-inflammatory NF-κB pathway [24]. Furthermore, cynaropicrin exhibits various metabolic regulatory activities, such as choleretic and anti-hyperlipidemic properties, thus supporting digestive health and fat metabolism [25]. It shows a broad spectrum of pharmacological actions, including anti-trypanosomal, anti-malarial, antifeedant, antispasmodic, anti-aging, anti-tumor and anti-Hepatitis C properties [26,27,28].

In this context, the comprehensive analysis and characterization of biowaste can enable the valorization of their bioactive molecules. Indeed, these matrices demonstrated diverse biological activities such as antimicrobial, antifungal, anticancer, and skin-protective functions [19,29,30]. This approach not only mitigates waste generation but also enhances the capacity of edible plant materials to exert positive effects on human, animal, and ecosystem health. By utilizing these bioactive properties, agricultural byproducts can be transformed into valuable resources, thereby advancing sustainability and delivering health benefits across various sectors.

Therefore, the aim of this study is to characterize and explore the biological activities of Tema cultivar biowaste to further increase its industrial value. The waste material was collected over the harvesting period and underwent a comprehensive chemical characterization to determine its concentration in its main bioactive molecules. Subsequently, the study investigates the waste’s efficacy on pivotal features of skin aging, namely wound healing (cell migration) and cellular senescence.

Through the chemical characterization and biological evaluation of artichoke waste, the authors attempt to unveil, for the first time, an innovative process for the sustainable use of agricultural byproducts of Tema cultivar, thus presenting new opportunities for a sustainable environmental and economic management of natural resources.

## 2. Results

### 2.1. Determination of Dicaffeoylquinic Acid Derivatives, Flavonoids, and Cynaropicrin

The HPLC-DAD analysis allows a comprehensive analysis of caffeoylquinic acid derivatives, flavonoids, and cynaropicrin across the harvesting season from November 2021 (batch A) to February 2022 (batch H). Eleven compounds were identified based on the Relative Retention time versus chlorogenic acid (Table 1). Chlorogenic acid and derivatives were quantified on the chlorogenic acid calibration curve, while the detected flavonoids were quantified on the cynaroside calibration curve. The spectrum types of the two compounds were type 1 for chlorogenic acid and type 2 for cymaroside (Figure S1). A total of six phenolic acids and four flavonoids were identified. Moreover, cynaropicrin was detected at 205 nm, which is a sesquiterpene lactone that shows an immunomodulatory effect and a protective action against certain pathogens and stimulates bile and fat metabolism [24,25,26,27,28]. Cynaropicrin was detected by the RRt versus cynaroside, whose calibration curve was used for the quantification. The quantification from batch A to H is reported in Table 2.

The concentrations of the detected compounds were monitored during the harvesting period (from batch A to H), and the corresponding concentrations in mg/g are reported in Table 2. Among the compounds analyzed, the phenolic acids were the most abundant throughout the collecting period, specifically chlorogenic acid, 3,4-Di-*O*-caffeoylquinic acid, and 3,5-Di-*O*-caffeoylquinic acid.

Chlorogenic acid showed a significant increase from 1.52 mg/g in the first half of November (A) to a peak of 7.48 mg/g in the first half of January (E) before slightly declining to 5.91 mg/g at the end of February (H).

3,4-Di-*O*-caffeoylquinic acid followed a similar trend, starting at 1.60 mg/g in batch A, peaking at 8.89 mg/g in batch G, and then decreasing to 5.72 mg/g in batch H. 3,5-Di-*O*-caffeoylquinic acid also increased from 2.19 mg/g in batch A to a maximum of 3.66 mg/g in batch E, maintaining relatively high levels until the end of February (3.12 mg/g). Cynarin and scolymoside exhibit a similar trend with lower concentrations; both showed a fluctuating pattern, peaking in the first half of January (E) until the first half of February (G), at concentrations of 0.48 and 0.18 mg/g, respectively. Cynaroside exhibits its peak slightly later, in the February batches G-H.

Low quantities of cynaropicrin were found in the samples, indicating an inconsistent trend over the harvesting period. It was detected in the initial samples, with the highest concentration observed in batch B at 405.4 µg/g, while it was absent in the later samples except for batch G, where it showed the second-highest concentration at 153 µg/g. Additionally, it presents a higher variability within the same batch, as indicated by the higher standard deviation.

With the cynaropicrin exceptions, the pattern found suggests a general compound increase as the season progresses toward winter, peaking in January. The data indicate an accumulation during the harvest season, possibly in response to environmental stress factors or as part of the plant’s maturation and metabolic processes.

### 2.2. Principal Component Analysis (PCA)

**Figure 1:**
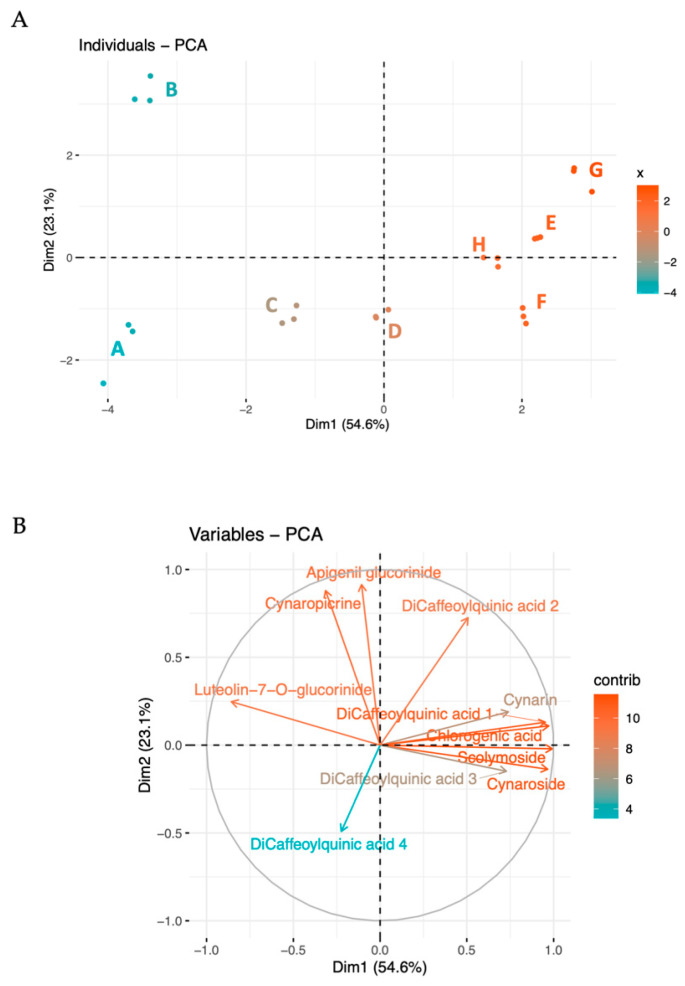
Biplot and PCA analysis of the detected compounds in the globe artichoke agro-wastes, from batches A to H. In the biplot (**A**), each dot represents a batch of replicates. The PCA (**B**) indicates the variable contribution. Colors tending toward red indicate better-represented variables, while gradually lighter colors toward blue shades indicate less-represented variables.

The metabolites were further analyzed using multivariate techniques, including Principal Component Analysis (PCA), showed in Figure 1. The first two principal components explain a significant portion of the variance (54.6% and 23.1%, respectively), indicating clear metabolic profiles associated with different collection periods (Figure 1A). Dimension 1 (PC1) effectively differentiated the batches according to their collection time. This indicates that the primary source of variation captured by Dimension 1 is closely related to the temporal changes in metabolite concentrations, reflecting seasonal influences or maturation stages.

Dimension 2 (PC2) contributed to further differentiation, explaining a smaller yet significant proportion of the total variability. In particular, PC2 focuses on metabolites that deviate from the main seasonal trend, such as apigenin and cynaropicrin, capturing less dominant differences, such as microenvironmental differences or unique responses to biotic or abiotic stressors.

The loading of variables (Figure 1B) further explains the most characterizing variables in the two principal dimensions. As previously highlighted, the most abundant compounds—chlorogenic acid, 3,4-di-O-caffeoylquinic acid, cynaroside, and scolymoside—exhibit high loadings and contribute to PC1. The prominence of these compounds in the loading plot confirms their substantial impact on the overall data structure and highlights their relevance in driving the main trends observed in the dataset.

PC2 is distinguished by variables such as apigenin, cynaropicrin, 1,5-di-*O*-caffeoylquinic acid, and 4,5-di-*O*-caffeoylquinic acid, with apigenin and cynaropicrin providing a more substantial contribution. This differential contribution of PC2 explains the separation between batches B and A. In fact, B exhibits higher levels of cynaropicrin and 1,5-di-*O*-caffeoylquinic acid, whereas sample A shows lower values for these compounds. This distinction highlights how PC2 captures unique variations not accounted for by the main seasonal trend, illustrating specific deviations in metabolite profiles that could be driven by physiological or environmental conditions affecting these metabolites.

Together, these dimensions provide a comprehensive view of how the metabolite profiles vary with both time and additional influencing factors, thus offering deeper insights into the complex dynamics of metabolite synthesis and accumulation.

Based on quantification data and multivariate analysis, specific batches have been chosen to assess the biological potential of the average sample. Batch A, representing the beginning of the collection, along with batches E and F, representing the second half of the collection and the beginning of the increasing trend, have been selected.

### 2.3. Biological Activities

#### 2.3.1. Effect of Cynara Agro-Wastes on Cell Viability

**Figure 2:**
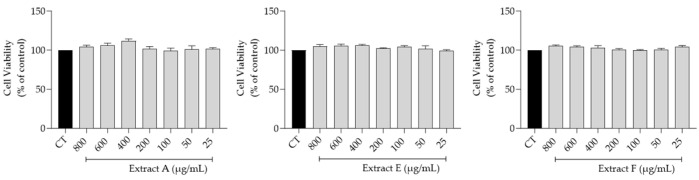
Safety profile of the hydroalcoholic extract from globe artichoke agro-wastes (batches A, E, and F) on fibroblasts assessed by the resazurin assay.

The effect of the hydroalcoholic extracts obtained from the three selected batches (A, E, and F) on fibroblasts (3T3 cell line) viability, a main target for skin anti-aging therapy, was assessed. Overall, as shown in Figure 2, no toxicity was observed at the tested concentrations (800–25 μg/mL), thus highlighting a very safe profile for all the extracts.

#### 2.3.2. Effect of Cynara Byproducts on Cell Migration

**Figure 3:**
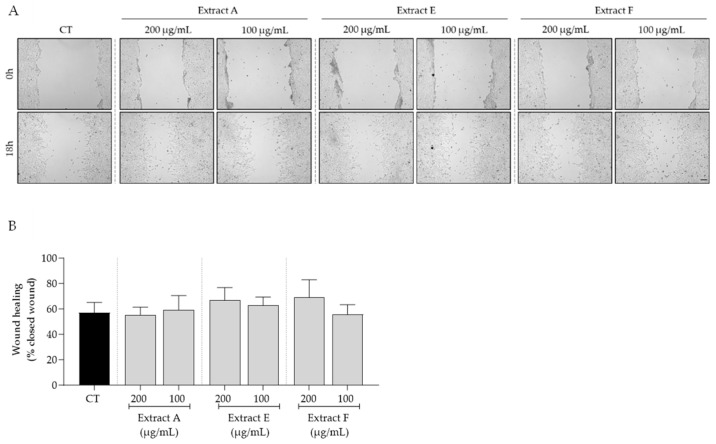
Effect of hydroalcoholic extracts from globe artichoke agro-wastes (batches A, E, and F) on fibroblasts migration assessed by the wound healing assay. (**A**) Images acquired by phase-contrast microscopy immediately after wound induction (0 h) and after 18 h of incubation; (**B**) histograms showing the quantified wound area of the different extracts compared with the control (CT). Scale bar: 100 µm.

Bearing in mind the overall safe profile observed for the tested extracts, we selected the intermediate concentrations of 100 and 200 μg/mL to further assess the bioactive potential of these byproducts on features related to skin aging. First, the effect of the extracts on cell migration, a feature highly compromised in aged skin, was tested. As shown in Figure 3, no significant effects on fibroblast migration were observed, although samples E and F at 200 μg/mL showed a slight tendency to increase this feature (Figure 3A,B).

#### 2.3.3. Effect of Cynara Byproducts on Cellular Senescence

**Figure 4:**
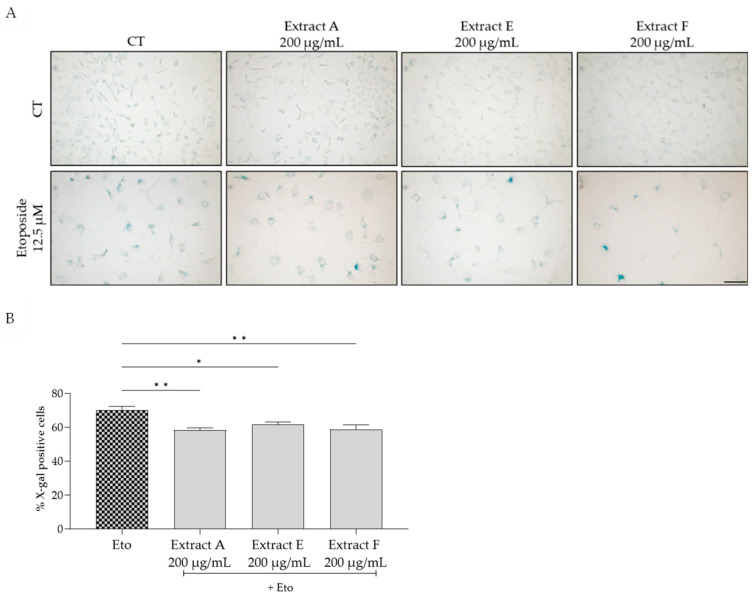
Effect of the hydroalcoholic extracts obtained from globe artichoke agro-wastes (batches A, E, and F) on NIH/3T3 fibroblasts senescence-associated β-galactosidase activity (**A**). Histograms showing the quantitative analysis using ImageJ software (**B**). Statistical significance * *p* < 0.05, ** *p* < 0.01 determined by one-way ANOVA followed by Dunnett’s multiple comparisons test. Eto—etoposide (12.5 µM); Scale bar: 100 µm.

To assess the effect of the extracts on cellular senescence, three complementary markers were considered, related to lysosomal activity, cell cycle arrest, and DNA damage. Regarding the first, senescence-associated (SA) β-galactosidase activity was determined in NIH/3T3 fibroblasts. The senescence inducer etoposide was used to induce an increase in this marker, as observed in Figure 4A,B. Interestingly, all the tested extracts, at 200 µg/mL, significantly inhibited this increase, pointing out a potential anti-senescent effect of the extracts, with extracts A and F being more effective (Figure 4B).

**Figure 5:**
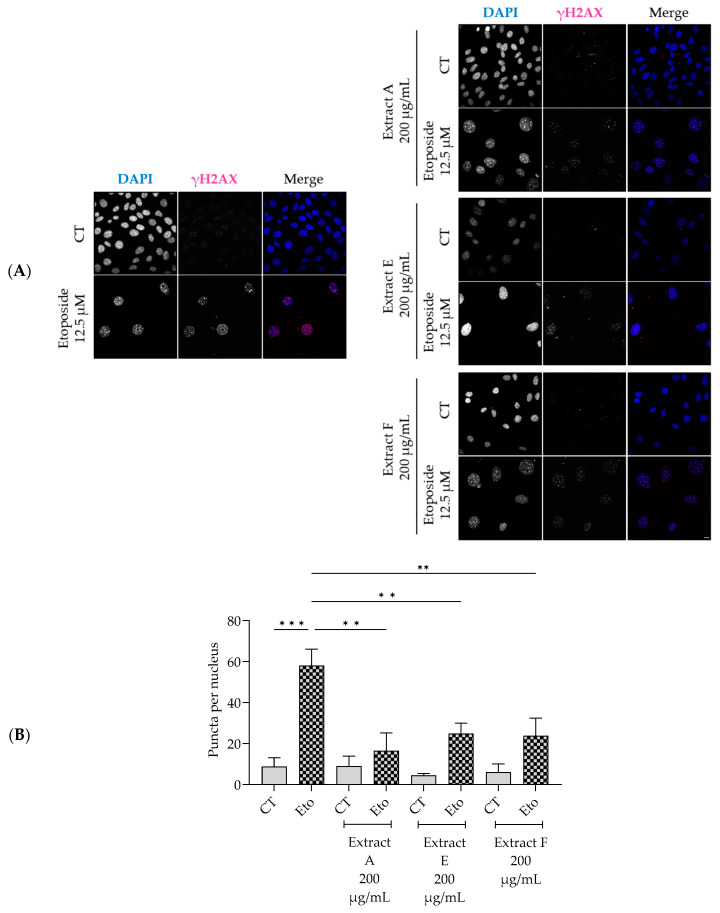
Effect of the hydroalcoholic extracts from globe artichoke agro-wastes (batches A, E, and F) on NIH/3T3 fibroblasts nuclear accumulation of the phosphorylated form of H2AX (γ-H2AX). Representative confocal images (**A**) and corresponding histograms from quantitave analysis (**B**). Statistical significance ** *p* < 0.01, *** *p* < 0.001 by one-way ANOVA followed by Dunnett’s multiple comparisons test. Eto—etoposide (12.5 µM); Scale bar: 10 µm.

Similarly, the extracts significantly decreased the phosphorylation and nuclear accumulation of γH2AX induced by etoposide (Figure 5A,B), thus pointing out a protective effect on double-strand DNA damage.

**Figure 6:**
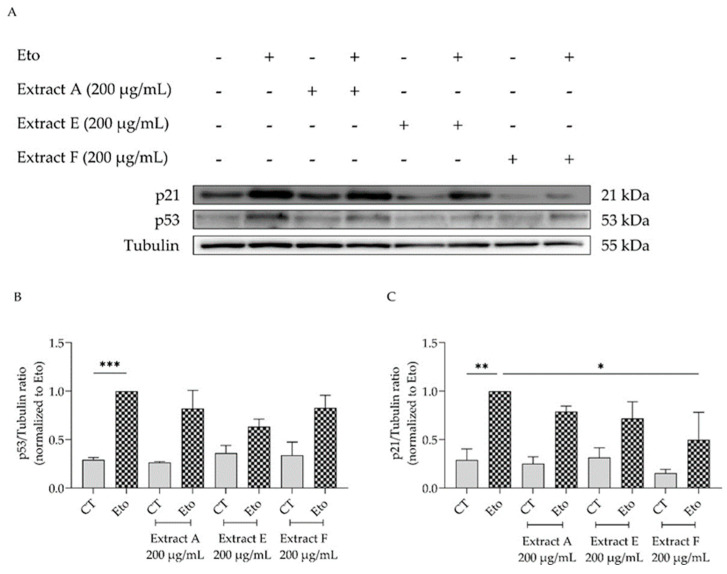
Effect of the hydroalcoholic extracts from globe artichoke agro-wastes (batches A, E, and F) on protein levels of p53 (**A**,**B**) and p21 (**A**,**C**). Tubulin used as loading control and results normalized to etoposide. * *p* < 0.05, ** *p* < 0.01, *** *p* < 0.001 by one-way ANOVA, followed by Dunnett’s multiple comparison test. Eto—etoposide (12.5 µM).

To corroborate these results, the effect of the extracts on cell cycle arrest was determined by evaluating their capacity to modulate the p53/p21 signaling pathway (Figure 6), which plays a key role in the initiation of senescence. Overall, the extracts show a tendency to decrease the protein levels of both p53 (Figure 6A,B) and p21, with extract F attaining statistical significance for the latter (Figure 6A,C).

## 3. Discussion

In the present work, the authors provide a characterization of the variation of the main phenolic acids, flavonoids, and cynaropicrin of Tema globe artichoke biowaste in relation to harvest time. Moreover, aiming at a potential valorization of these wastes, their protective effect against skin aging was investigated, pointing out novel applications in the cosmeceutical or pharmaceutical fields. Firstly, the outer bracts of the Tema cultivar were selected as biowaste. This selection was driven by two factors: the amount of available waste and the location of the collection. While some organic waste is typically left in the field (e.g., secondary or tertiary head flowers), the outer bracts of this cultivar are processed on the farm as part of the fourth-range processing, therefore simplifying the collection operations [7]. The information available in the literature reports different advantages of the Tema cultivar: Lombardo et al. [5] found the highest number of polyphenols in Tema out of the other seventeen artichoke cultivars tested. Accordingly, a high polyphenolic content was found by Cabezas-Serrano et al. [6], which, however, highlighted a low vitamin C content and a related low antioxidant activity. This cultivar is also sufficiently resistant to browning, making it suitable for processed products and rendering its waste particularly amenable to further utilization [6].

The chemical characterization here performed unveiled 11 main compounds belonging to the classes of phenolic acids and derivatives of caffeoylquinic acids, flavonoids, and the sesquiterpene lactone cynaropicrin. Based on the literature, these bioactive molecules are typically more abundant in the heart of the artichoke [31]. In contrast, higher values were found here when compared to those typically found in the outer bracts, [30], which differences may be attributed to the characteristics of this hybrid cultivar.

From a quantitative point of view, the most abundant compounds were chlorogenic acid (7.14 mg/g in Batch E) and 3,4-di-*O*-caffeoylquinic acid (8.89 mg/g in Batch G). Followed by 3,5-di-*O*-caffeoylquinic acid, scolymoside, and cynaroside. Chlorogenic acid, the most abundant compound found here and typically abundant in artichoke, is reported to have potent antioxidant and anti-inflammatory activities [22,29,32].

In addition, the analysis of the biowaste during the harvesting period underscores a significant variation in polyphenol content. This parallels prior findings on the content of chlorogenic acid in other crops and their byproducts, including potato [33], faba bean [34], and tomato [35]. In particular, it was interesting to note a visible trend for the majority of compounds, resulting in an increase in concentration in the late harvest lots. Specifically, this was evident in 7 out of 11 compounds, leading to an overall increase of 15.13 mg/g for chlorogenic acid derivatives and 1.30 mg/g for cynaroside derivatives. Multivariate analysis clearly highlighted this phenomenon. It clusters the batches based on harvest time in the first dimension and accounts for 54% of the variability. Many studies investigate artichoke’s polyphenol variation among different cultivars and different parts of the plant (e.g., leaves, head, inner and outer bracts), while little is known about phenol trends during artichoke seasonality [5,6,11,19]. Lombardo et al. (2010) demonstrated that Romanesco artichokes exhibit higher phenol content in the spring harvest compared to the winter harvest, with a slight increase in the outer bracts and a pronounced increase toward the inner parts of the flower head, reaching a maximum in the floral stem (values up to 16 times higher). Moreover, various forms of stress, such as drought, are known to elevate artichoke polyphenol levels [36]. Therefore, the increased polyphenol content can be attributed to progressive lignification during maturation, as well as to biotic and abiotic stresses, in order to enhance physical resistance: these polyphenols, including chlorogenic acids and its derivatives, are part of the phenylpropanoid pathway, which are utilized for lignin production in the outer bracts, whereas in the innermost portions, they remain more abundant as precursors [30].

Cynaropicrin, a sesquiterpene lactone characterized by a tricyclic structure featuring a butyrolactone ring, did not show this seasonal pattern: its concentration varied widely across different batches (the highest concentration was found in batch B, at 405 µg/g, while it was absent in late batches except for G, at 153.34 µg/g). This observation aligns with the literature, which reports higher levels of cynaropicrin in the leaves [37]. In contrast, it is typically present in low quantities in the inflorescence receptacle at the earliest stages of development, while it disappears at more advanced stages (0.05 mg/g when reaching the commercial stage) [25]. In contrast, the literature reports no cynaropicrin detection in the outer bracts. This may indicate a difference in cultivar characteristics; for example, Tema is a particularly tender cultivar suitable for raw consumption, whereas Romanesco, as tested by Eljounaidi et al. [25], is generally larger and fibrous, then suitable for cooked consumption.

In particular, in-vitro tests have demonstrated antiproliferative effects at very low concentrations (5, 7.5, and 10 micrograms per milliliter) against colorectal cancer cells [38]. Additionally, there is evidence of activity against lung, multiple myeloma, and melanoma cancer cell lines [39,40,41]. Based on the analyses performed, the outer bracts could still be a potential target for the industrial recovery of natural bioactive compounds to replace synthetic ones.

The bioactive studies focused on the anti-aging potential of the hydroalcoholic extracts of three selected batches (A, E, and F), particularly on features related to skin aging. Overall, significant anti-senescence effects were observed, thus highlighting a potential health-promoting effect for these agro-wastes. Indeed, particularly for extract F, one of the extracts representing the second half of the harvest period, significant inhibition was attained in all senescence complementary markers, namely in lysosomal activity (β-galactosidase activity), in cell cycle arrest (p53/p21 pathway) and in DNA damage (nuclear accumulation of γH2AX). *Cynara scolymus* is known for its health benefits due to its high polyphenolic content, as recently reviewed by Porro and colleagues [42] that pointed out anti-inflammatory, antioxidant, liver-protective, bile-expelling, antimicrobial, and lipid-lowering neuroprotective effects. Although the artichoke biowastes showed a tendency to promote wound healing here, this effect has been shown for other species of the genus. For example, an ointment made with *C. humilis* powder extract was able to increase neovascularization, collagen deposition, and re-epithelialization in an animal model of skin burn [43]. Moreover, in a different study, the wound healing effects of both ethanolic and aqueous extracts of this species promoted wound surface recovery, re-epithelization, and collagen deposition, with a concurrent decrease in wound scarring [44].

Regarding the anti-senescence effects of globe artichoke biowaste herein reported, the results are in line with a previous study that also highlighted the potential of an artichoke extract on skin anti-age effects, although resorting to a different approach. This study focused on the effect of the extract on endothelial cell integrity and functionality [45]. In addition, Mileo and colleagues showed that long-term exposure to polyphenols from artichoke induced cell cycle arrest, as indicated by an increase in p16 and p21 expressions [46]. Although these results seem contradictory, the study was performed on a human breast cancer cell line, in which inducing senescence is beneficial to avoid cell proliferation, thus pointing out a potential application in cancer prevention/treatment.

The main compounds present in artichoke biowaste, namely chlorogenic acid and their derivatives as well as cynaroside, also gather relevant bioactive potential, namely in what concerns delaying skin aging. For example, it was shown that 4,5-dicaffeoylquinic acid [47] and chlorogenic acid [48] promote wound healing. In addition, the presence of 3,5-dicaffeoylquinic acid ameliorated spatial learning and memory in a senescence-accelerated-prone-mice model [49], suggesting that this compound might have anti-senescent properties. Moreover, chlorogenic acid and several dicaffeoylquinic acids increased the lifespan of *C. elegans*, with 3,5-dicaffeoylquinic acid downregulating the insulin/insulin-like growth factor signaling (IIS) pathway [50]. Interestingly, this pathway seems to be responsible for the lifespan-enhancing properties of cynaroside (luteolin-7-*O*-glucoside) [51], a compound present in all agro-waste batches tested. This compound, in a model of UVA-induced photoaging on keratinocytes, decreased metalloproteinase-1 (MMP-1) production by modulation of the mitogen-activated protein kinases (MAPKs) and activator protein-1 (AP-1) signaling pathways [52], thus reinforcing its protective effect. Another relevant compound in artichoke is cynaropicrin. This compound is known to suppress skin photoaging by inhibiting nuclear factor-kappa B transcription activity [53], as well as the AhR-Nrf2-Nqo1 pathway [54]. However, this compound is not present in sample F and, therefore, does not contribute to the anti-skin aging effects herein reported.

## 4. Materials and Methods

### 4.1. Biowaste Material: Collection and Preparation

This work is based on the collection of the biowaste generated from the production chain of *Cynara scolymus* L. “Tema” cultivar from the fall-winter 2021–2022 harvest (Table 3). The artichokes were harvested from the field and processed for the fresh market (fourth-range products).

In brief, artichokes intended for the fourth-range market (Figure S2) were prepared by removing the outermost bracts from the artichoke’s head. The processed artichokes were subsequently treated with antioxidant solutions or citric acid to reduce microbial load, inhibit enzymatic browning, and extend shelf life. This product is primarily allocated to the large-scale retail trade (GDO). The outer bracts, separated from the capitula during initial processing, represent the waste material and are discarded. The Tema cultivar was specifically selected due to its predominant use as a fourth-range product, wherein processing generates a substantial amount of waste (bracts) on the farm, simplifying harvesting operations.

The wastes (bracts) were recovered constantly every 15 days during the entire production season of the Tema cultivar, starting in November 2021 to the end of February 2022 (Table 3) following the operational activities of a company in the middle Campidano area of Sardinia region, Italy (coordinates: Samassi 56 m asl., 39°28′53.48″ N 8° 54′19″ E). The biowaste was collected in the farm and transferred to the laboratory, weighed and oven-dried (FD 115, BINDER) at 40 °C, until complete water removal. It was then subjected to grinding with an electric grinder and vacuum-stored for further processing.

### 4.2. Biowaste Extraction

The extraction followed the methods proposed by Gonçalves et al. [55] with some modifications. Briefly, two grams of dry bracts were extracted overnight in constant agitation by a hydroalcoholic solution EtOH-H_2_0 70/30 (*v*/*v*) at 70 °C with a total volume of 250 mL. The extract was filtered through Whatman N.1 filter paper, following the ethanol remotion using a rotary vacuum evaporator at 40 °C. For better preservation, each extract was frozen and freeze-dried to completely remove the water (LIO 5PDGT lyophylizer, Cinquepascal S.r.l., Trezzano, Italy, with nXDS6i pump, Edwards Limited, Burges Hill, UK). The obtained extracts were redissolved in methanol (HPLC-grade), filtered using 0.45 µm filters, and then analyzed. The extraction was performed in triplicate.

### 4.3. HPLC-DAD Analysis

HPLC analysis was performed using an HPLC chromatography system (1260, Agilent Technologies, Santa Clara, CA, USA) equipped with a quaternary pump, a diode array detector operating at 263 nm and 203 nm, and an auto-injector. Separation was achieved using a water-reversed phase column (Zorbax Eclipse XDB-C18 RaPID Res 250 × 4.6 mm I.D.) at 20 °C. The mobile phase comprised water (Solvent A) and acetonitrile (Solvent B), with 0.01% of Trifluoroacetic Acid (TFA). The used gradient program is reported in Supplementary Materials (Table S1). The chromatographic run was performed at room temperature, at 1.0 mL/min of flow rate, and 20 µL of sample were injected. The quantification of dicaffeoylquinic acid derivates and flavonoids was obtained according to Schütz et al. [56], using chlorogenic acid and cynaroside as external standards, with UV detection at 263 nm. The quantification of cynaropricrin was obtained using cynaroside as an external standard, with UV detection at 205 nm [57].

Chlorogenic acid (Merck Reference Standard) and Cynaroside (Luteolin 7-*O*-glucoside − Merck analytical standard) were used as standards and for the preparation of the stock solutions (respectively with the concentrations of 0.16 mg/mL and 0.06 mg/mL). Several dilutions from the stock solutions were prepared by the addition of MeOH. All the standard dilutions were then stored at 4 °C and were stable for at least 30 days. Calibration curves were obtained by comparing the concentration with respect to the response of the peak area (Figure S3).

### 4.4. Cell Culture

NIH/3T3 fibroblasts, purchased from the American Type Culture Collection (ATCC CRL-1658), were cultured as previously described [58]. Briefly, Dulbecco’s Modified Eagle’s Medium (DMEM, Gibco, Thermo Fisher Scientific, Waltham, MA, USA, Ref 31600-083) containing 25 mM glucose, 3.7 g/L sodium bicarbonate, 100 U/mL penicillin, and 100 μg/mL streptomycin and heat-inactivated fetal bovine serum (FBS, 10%) was used. The cells were subcultured upon reaching 70–80% confluency, and their morphology was monitored using an inverted light microscope.

### 4.5. Cell Viability

The effect of the selected hydroalcoholic extracts from globe artichoke agro-wastes on cell viability was examined using the resazurin reduction assay, as previously reported [59]. In summary, NIH/3T3 fibroblasts were seeded at a density of 50,000 cells/mL in 48-well plates and allowed to stabilize overnight. After 24 h of exposure to the extracts (800 to 25 µg/mL), the medium was discarded, and a fresh medium with resazurin (1:10) was added for 2 h. Absorbance was measured at 570 nm using a reference filter of 620 nm on an automated plate reader (SLT, Salzburg, Austria).

### 4.6. Cell Migration

The scratch wound assay based on the method described by Martinotti and Ranzato [60] with slight modifications was used. NIH/3T3 fibroblasts were plated at 300,000 cells/mL in 12-well plates and incubated for 24 h. Then, a scratch was induced in the cell monolayer using a pipette tip. The detached cells were removed by washing with sterile PBS, and cells were maintained in a culture medium supplemented with 2% FBS, with or without the extracts (200 or 100 μg/mL). Phase-contrast microscopy was used to capture images immediately after wound induction and following 18 h of incubation, and the area of the wound was quantified using an ImageJ/Fiji plugin [61].

### 4.7. Anti-Senescence Potential

#### 4.7.1. Senescence-Associated β-Galactosidase Activity

Etoposide was used to induce senescence in NIH/3T3 fibroblasts [62] seeded at 15,000 or 30,000 cells/mL for control or etoposide-treated cells, respectively. Cells were allowed to stabilize overnight, and then etoposide (12.5 μM) was added for an additional period of 24 h. Subsequently, etoposide was removed, and cells were further incubated for 24 h with or without the extracts (200 µg/mL). Beta-galactosidase activity was detected with a commercial kit according to the manufacturer’s instructions (#9860, Cell Signalling Technology Inc., Danvers, MA, USA). Cells were imaged for quantitative analysis using ImageJ software (https://imagej.net/ij/), with senescent cells presenting a green stain, indicative of β-galactosidase activity, a known characteristic of senescent cells.

#### 4.7.2. Nuclear Staining of Histone γH2AX

NIH/3T3 were cultured on glass coverslips, treated as reported in Section 4.7.1, and fixed for 15 min with 4% paraformaldehyde (PFA), followed by three washing steps with sterile PBS. 0.1% Triton X-100 for 15 min was used for permeabilization, followed by three washing steps with PBS. Then, cells were incubated with a blocking solution (3% bovine serum albumin and 10% goat serum in PBS) for 1 h. γH2AX was detected using a primary antibody (1:500, Cell Signalling 9718) applied to cells overnight at 4 °C. Afterward, cells were washed three times with PBS and incubated for 1 h at room temperature with a secondary antibody (1:500, goat anti-rabbit Alexa Fluor 564) and DAPI (1:2000). Following washes with PBS, coverslips containing the cells were mounted on glass slides with Mowiol mounting medium. Images were obtained using a confocal point-scanning microscope (Zeiss LSM710; Carl Zeiss, Jena, Germany) with a 63× objective.

#### 4.7.3. p21 and p53 Protein Levels

NIH/3T3 fibroblasts cultured at 200,000 and 400,000 cells/mL were seeded in 6-well plates and treated as described in Section 4.7.1. Then, cell lysates were prepared, and protein separation and immunoblotting were carried out as previously established [63]. Post-blocking, membranes were incubated at 4 °C overnight with specific antibodies against p21 (1:1000, Abcam ab188224, Waltham, MA, USA) and p53 (1:1000, Proteintech 10442-1-AP, Chicago, IL, USA). Then, membranes were washed with TBS-T for 10 min, three times, and incubated for 1 h at room temperature with horseradish peroxidase-conjugated secondary antibodies (1:20,000). Detection of proteins was performed using a chemiluminescence scanner (Image Quant LAS 500, Cytiva, Tokyo, Japan). Tubulin (1:20,000; Sigma, St. Louis, MO, USA) served as a loading control, and ImageLab software version 6.1.0 (Bio-Rad Laboratories Inc., Hercules, CA, USA) was used for protein quantification.

### 4.8. Statistics

Statistical analyses were performed using R software version 2023.06.1+524 [64] and the packages agricolae, car, and ggplot2. All data were initially tested for normal distribution and homogeneity of variances, using the Shapiro–Wilk test for normality and Levene’s and Bartlett’s test for analysis of variance, respectively. Based on the results, parametric or non-parametric tests were used for the chemical profile analysis (ANOVA fb post-hoc Tukey’s test for parametric data; Krustal–Wallis fb Wilcoxon test for non-parametric data). Data visualization (PCA) was obtained using R software, with the packages ggplot2 and ggpubr.

Results regarding globe artichoke agro-waste bioactivities were conducted in three independent experiments, performed in duplicate. Results are presented as mean ± SEM (standard error of the mean) values. Statistical significance was evaluated by one-way analysis of variance (ANOVA) followed by a suitable post-hoc analysis using GraphPad Prism 9.3.0 software. *p* values below 0.05 were considered statistically significant.

## 5. Conclusions

The present study not only provides the chemical characterization of the Tema cultivar but also provides, through the evaluation of its agro-waste biological activity, a useful insight into its valorization. The application of the agroprospecting concept involved a crucial step of analyzing the crops’ production chain and their derived biological waste. In this case, the main advantage was the choice of an artichoke intended for sale as a fourth-range product, for which on-farm processing allows simplified waste collection.

Chemical characterization provided confirmation of phenolic acid and flavonoid content, while seasonal monitoring revealed their variability during the harvest season. For this reason, biological tests covered early and late harvest samples, which reveal similar action in wound healing, while greater efficacy of late batch F in antisenescence action highlights the potential anti-aging effects. These findings reveal the need for analysis of agro-waste during seasonality to make better utilization (e.g., as a supplement, human food or animal feed, or pharmaceutical or nutraceutical products).

In conclusion, this approach aims to foster a truly sustainable circular economy. An intriguing direction for future research is to determine how chemical composition varies in response to different factors such as cultivation environment, crop management practices, industrial processing, and marketing, all of which can affect the quality of biomolecules in these crop species. Furthermore, the same concept can easily be exported to different types of crops and their corresponding agro-wastes.

## Figures and Tables

**Table 1:** Identified compounds in test solution chromatogram by means of Relative Retention times (RRt) versus chlorogenic acid (263 nm) and cynaroside (205 nm).

Compounds	Approximate Retention Time ^a^	RRt versus Chlorogenic Acid	Spectrum Type	Quantified as
Chromatographic run at 263 nm				
Chlorogenic acid	16.7	1.00	1	Chlorogenic acid
Cynarin	21.0	1.26	1	Chlorogenic acid
Scolymoside	23.3	1.40	2	Cynaroside
Cynaroside	24.5	1.47	2	Cynaroside
Luteolin-7-*O*-glucorinide	24.8	1.49	2	Cynaroside
3,4-Di-*O*-caffeoylquinic acid	26.4	1.58	1	Chlorogenic acid
1,5-Di-*O*-caffeoylquinic acid	27.7	1.66	1	Chlorogenic acid
3,5-Di-*O*-caffeoylquinic acid	28.2	1.69	1	Chlorogenic acid
Apigenil glucorinide	29.3	1.75	2	Cynaroside
4,5-Di-*O*-caffeoylquinic acid	30.3	1.81	1	Chlorogenic acid
Chromatographic run at 205 nm				
Cynaroside	25.1	1.00	2	Cynaroside
Cynaropicrin	38.3	1.53	2	Cynaroside

^a^ Retention Time expressed in min.

**Table 2:** Quantification of the phenolic acids (in green), flavonoids (in orange), and cynaropicrin (in yellow), identified in the hydroalcoholic extracts of Tema biowaste, from harvesting batches from November (A) to February (H).

Compounds	Quantified as	A	B	C	D	E	F	G	H
Chlorogenic acid ^1^	Chlorogenic acid 263 nm	1.52 ± 0.05 *	2.14 ± 0.11 *	2.31 ± 0.08 *	3.76 ± 0.03 *	7.48 ± 0.07 *	6.98 ± 0.02 ^a^	7.14 ± 0.03 ^a^	5.91 ± 0.01 *
Cynarin ^1^		0.09 ± 0.01 ^cd^	0.08 ± 0.00 ^d^	0.06 ± 0.01 ^d^	0.08 ± 0.01 ^d^	0.18 ± 0.00 ^d^	0.12 ± 0.00 ^ab^	0.14 ± 0.01 ^a^	0.11 ± 0.01 ^bc^
3,4-Di-*O*-caffeoylquinic acid ^1^		1.60 ± 0.04 *	2.98 ± 0.02 *	3.37 ± 0.03 *	6.48 ± 0.08 *	8.12 ± 0.01 *	7.57 ± 0.04 *	8.89 ± 0.04 *	5.72 ± 0.02 *
1,5-Di-*O*-caffeoylquinic acid ^1^		0.63 ± 0.01 *	1.40 ± 0.02 ^a^	1.13 ± 0.01 *	1.04 ± 0.01 *	1.63 ± 0.00 *	0.82 ± 0.02 *	1.84 ± 0.00 *	1.38 ± 0.00 ^a^
3,5-Di-*O*-caffeoylquinic acid ^1^		2.19 ± 0.08 ^a^	2.04 ± 0.05 ^b^	2.06 ± 0.04 ^b^	2.91 ± 0.00 *	2.26 ± 0.00 ^a^	3.66 ± 0.01 *	3.27 ± 0.01 *	3.12 ± 0.00 *
4,5-Di-*O*-Caffeoylquinic acid ^1^		0.11 ± 0.19 ^a^	0.02 ± 0.02 ^a^	-	0.06 ± 0.00 ^a^	-	0.07 ± 0.01 ^a^	-	-
**Total Quantified as Chlorogenic Acid**		**6.15 ± 0.14**	8.67 ± 0.11 *	8.94 ± 0.15 *	14.33 ± 0.08 *	19.66 ± 0.08 *	**19.20 ± 0.07 ^a^**	**21.28 ± 0.03 ^a^**	16.25 ± 0.03 *
Scolymoside ^1^	Cynaroside - 263 nm	0.06 ± 0.01 ^b^	0.06 ± 0.01 ^b^	0.18 ± 0.00 *	0.21 ± 0.00 *	0.40 ± 0.00 ^a^	0.40 ± 0.00 ^a^	0.48 ± 0.00 *	0.37 ± 0.00 *
Cynaroside ^1^		0.30 ± 0.01 *	0.21 ± 0.01 *	0.56 ± 0.01 *	0.58 ± 0.01 ^a^	0.86 ± 0.00 ^a^	1.09 ± 0.01 *	1.15 ± 0.00 *	1.00 ± 0.00 *
Apigenil glucorinide ^1^		0.02 ± 0.00 ^a^	0.07 ± 0.01 *	-	-	-	0.03 ± 0.00 ^a^	0.04 ± 0.00 *	0.03 ± 0.00 ^a^
Luteolin-7-*O*-glucorinide ^1^		0.02 ± 0.00 ^a^	0.02 ± 0.00 ^a^	-	-	-	-	-	-
**Total Quantified as Cynaroside**		0.39 ± 0.00 *	0.35 ± 0.02 *	0.74 ± 0.01 *	0.79 ± 0.01 *	**1.26 ± 0.00 ^a^**	**1.52 ± 0.01 ^a^**	1.68 ± 0.00 *	1.40 ± 0.00 *
**Cynaropicrin ^2^**	Cynaroside 205 nm	55.51 ± 48.08 ^a^	405.2 ± 1.3 *	36.78 ± 12.63 ^a^	70.83 ± 2.6 ^a^	-	-	153.34 ± 38.0 *	-

^1^ Values represent the mean value of three experimental replicates ± standard deviation (SD), expressed in mg/g. ^2^ Values represent the mean value of three experimental replicates ± standard deviation (SD), expressed in μg/g. * Asterisk indicates statistical significance with respect to all comparisons tested, while the absence of statistical significance in the comparison between two groups is indicated by equal letters.

**Table 3:** Batches and corresponding harvest period.

Batch	Harvest Period (2021–2022)
A	1 November 2021
B	15 November 2021
C	1 December 2021
D	15 December 2021
E	1 January 2022
F	15 January 2022
G	1 February 2022
H	15 February 2022

## Data Availability

The original contributions presented in the study are included in the article/Supplementary Materials, further inquiries can be directed to the corresponding author/s.

## Supplementary Materials

The following supporting information can be downloaded at https://www.mdpi.com/article/10.3390/molecules29163960/s1, Figure S1: UV spectra of Chlorogenic Acid and Cynaroside, used for the quantification and identification (from Diode Array analysis). Figure S2: (a) Globe artichoke of Tema cultivar. (b) Tema fourth range product. Figure S3: (A) Cynaroside calibration curve; (B) Chlorogenic acid calibration curve. Table S1. Gradient programme of HPLC-DAD analysis.
