# Supplementary material for: Agroprospecting of Biowastes: Globe Artichoke (Cynara scolymus L. Cultivar Tema, Asteraceae) as Potential Source of Bioactive Compounds
Source: Molecules. 2024 Aug 22;29(16):3960. doi: 10.3390/molecules29163960 (PMC11356890; doi:10.3390/molecules29163960)
Supplement: Supplementary file 1 [file molecules-29-03960-s001.zip › molecules-3118288-supplementary.pdf]

Supplementary Materials

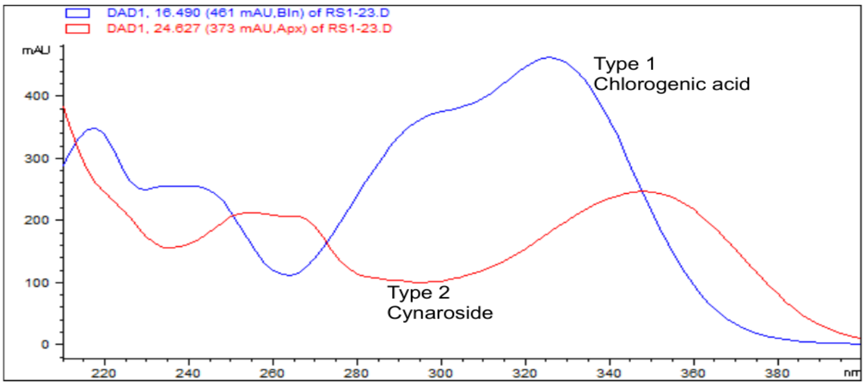

Figure S1: UV spectra of Chlorogenic Acid and Cynaroside, used for the quantification and identification (from Diode Array analysis).

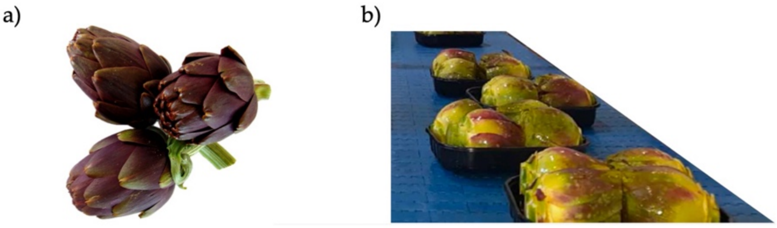

Figure S2: a) Globe artichoke of Tema cultivar. b) Tema fourth range product.

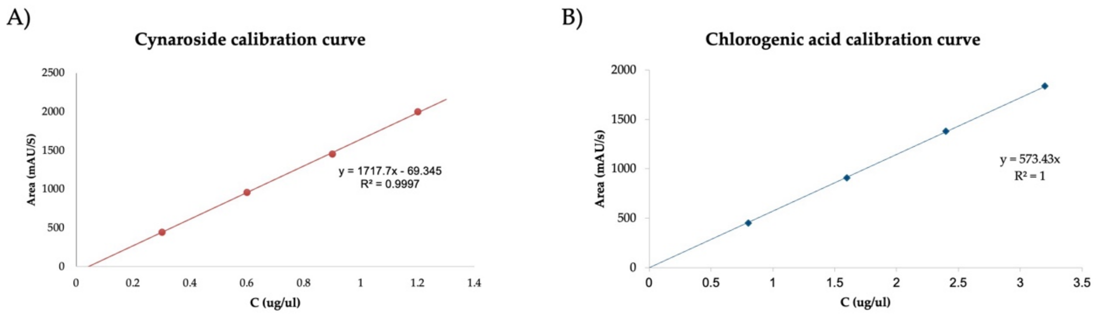

Figure S3: A) Cynaroside calibration curve; B) Chlorogenic acid calibration curve.

Table S1. Gradient programme of HPLC-DAD analysis

Time (min)[a]	Solvent A (%)[b]	Solvent B (%)[b]
0-4	95	5
4-44	78	22
44-49	65	35
49-50	20	80
50-60	95	5

[a] Time is referred to the starting time of the HPLC gradient. [b] Solvent A and B contained 0.001% of TFA.
